# The curious case of sporadic nematode susceptibility in “Tifguard” peanut (*Arachis hypogaea*): seed mixture or genetic instability?

**DOI:** 10.1093/g3journal/jkag175

**Published:** 2026-07-08

**Authors:** Samuele Lamon, Brian L Abernathy, Soraya C M Leal-Bertioli, David J Bertioli

**Affiliations:** Institute of Plant Breeding, Genetics and Genomics, University of Georgia, Athens, GA 30602, United States; Center for Applied Genetic Technologies, University of Georgia, Athens, GA 30602, United States; Institute of Plant Breeding, Genetics and Genomics, University of Georgia, Athens, GA 30602, United States; Center for Applied Genetic Technologies, University of Georgia, Athens, GA 30602, United States; Department of Plant Pathology, University of Georgia, Athens, GA 30602, United States; Institute of Plant Breeding, Genetics and Genomics, University of Georgia, Athens, GA 30602, United States; Center for Applied Genetic Technologies, University of Georgia, Athens, GA 30602, United States; Department of Crop & Soil Sciences, University of Georgia, Athens, GA 30602, United States

**Keywords:** peanut, *Arachis hypogaea*, “Tifguard”, root-knot nematode, wild introgression, disease resistance, susceptibility, symptomatic, asymptomatic, genetic instability

## Abstract

The Runner-type peanut (*Arachis hypogaea* L.) cultivar “Tifguard” carries an introgressed chromosomal segment on chromosome A09 from *A. cardenasii* that confers resistance to root-knot nematode (RKN). Despite this, a proportion of “Tifguard” plants show RKN symptoms, which could plausibly be attributed to seed mixture or outcrossing. However, recent work has shown that cultivated peanut exhibits surprisingly frequent large-scale chromosomal instability (1% to 5%); suggesting that resistance loss could arise from spontaneous structural genomic change. To test these possibilities, we grew foundation seed in an RKN-infested field and collected symptomatic and asymptomatic plants. Lineages derived by single-seed descent were genotyped using the Axiom *Arachis* 48K SNP array v2 and whole-genome sequencing. Symptomatic lineages lacked the *A. cardenasii* introgression on chromosome A09 and instead carried the complete endogenous *A. hypogaea* A09 region at the expected dosage. There was no evidence of large-scale homoeologous exchange, deletion, or other genomic instability affecting this chromosome. Most susceptible plants were closely related to resistant “Tifguard” but lacked the A09 introgression, with a smaller proportion assignable to known nematode-susceptible cultivars, implicating seed mixture with a possible contribution from cross-pollination rather than genomic instability. Because resistance depends on a single major-effect segment, rare events have disproportionate phenotypic impact, placing high demands on genetic purity. For important traits conferred by major loci, marker-based testing across seed-increase stages could verify trait retention directly, and is increasingly practical as marker costs decline.

## Introduction

Peanut (*Arachis hypogaea* L.; 2*n* = 4*x* = 40; AABB genome), a globally cultivated polyploid legume, is a valuable crop, with global production exceeding 50 million metric tons in recent years. The United States contributes approximately 2.9 million metric tons, with more than half of the production coming from Georgia (Bukowski and Ates 2023; FAO 2024). Peanut is valued for its high content of oil, protein, and antioxidants, making it a staple in diets worldwide, particularly in Africa and Asia (Stalker and Wilson 2015; Parilli-Moser et al. 2022).

The origin of peanut dates back less than 10,000 years, resulting from the spontaneous hybridization and polyploidization of 2 wild diploid progenitors: *A. duranensis* (2*n* = 2*x* = 20; AA genome) and *A. ipaënsis* (2*n* = 2*x* = 20; BB genome), which diverged around 2.5 million years ago (Bertioli et al. 2016, 2019). Hybridization and polyploidization produced fixed hybrid vigor, leading to larger plants and leaf cell sizes, and enhanced photosynthetic efficiency (Leal-Bertioli et al. 2012, 2017). However, polyploidization also caused a genetic bottleneck, isolating cultivated peanut from its wild relatives and limiting genetic and phenotypic diversity. This bottleneck has left peanut vulnerable to pests and diseases such as root-knot nematode (RKN) (caused by *Meloidogyne arenaria* Neal) (Motsinger et al. 1976; Ingram and Rodriguez-Kabana 1980; Wheeler and Starr 1987; Bertioli et al. 2019).

Wild *Arachis* sp. serve as a valuable reservoir of resistance to pests and diseases, and peanut breeders have refined the introgression of these traits into cultivated peanut as an effective strategy to address these vulnerabilities (Stalker 2017). For instance, wild species exhibit resistance to smut (caused by *Thecaphora frezii* Carranza & Lindquist) (Bressano et al. 2019), rust (caused by *Puccinia arachidis* Speg.) (Leal-Bertioli et al. 2010, 2015; Bertioli et al. 2021a, 2021b; Levinson et al. 2021), early and late leaf spot (caused by *Passalora arachidicola* [Hori] Braun and *Nothopassalora personata* [Berk. & M.A. Curtis] U. Braun, C. Nakash, Videira & Crous, respectively) (Bertioli et al. 2021a; Lamon et al. 2021; Gonzales et al. 2023), RKN (Simpson and Starr 2001; Leal-Bertioli et al. 2016; Ballén-Taborda et al. 2019, 2022; Bertioli et al. 2021a), and stem rot (caused by the fungus *Athelia rolfsii* [Curzi] C. C. Tu & Kimbr) (Bennett et al. 2021; Tsai et al. 2024). The introgression of these traits into cultivated peanut mitigates the effects of the genetic bottleneck, improving crop resilience and sustainability.

In the United States, *M. arenaria* race 1 is the primary RKN species affecting peanut, exhibiting a relatively narrow host range. It inflicts significant damage to peanut crops by reducing yields and degrading pod and seed quality, leading to stunted growth, plant death, and weakened pegs. In contrast, *M. arenaria* race 2, which has a broader host range, primarily affects vegetables and other crops but is not a major issue for peanut production (Osman et al. 1985). Traditional control methods of *M. arenaria* race 1, such as crop rotation and nematicides, remain limited due to environmental concerns. Thus, developing RKN-resistant peanut varieties offers a more sustainable solution (Starr et al. 2002).

“Tifguard,” a Runner-type peanut cultivar developed using a single-seed descent breeding scheme, is known for its resistance to both tomato spotted wilt virus (TSWV) and RKN (Holbrook et al. 2008). TSWV resistance originates from “C-99R” (Gorbet and Shokes 2002) while RKN resistance is conferred by a chromosomal segment on A09, derived from the diploid wild peanut relative *A. cardenasii* (Simpson et al. 1993; Nagy et al. 2010). This large segment was inherited from “COAN,” the first peanut variety developed with RKN resistance (Simpson and Starr 2001; Nagy et al. 2010).

“Tifguard” has been widely used in the development of several other peanut cultivars and germplasm lines. It is the direct parent of “TifNV-High O/L” (Holbrook et al. 2017), “Georgia-19HP” (Branch and Brenneman 2020), “FloRun ‘331’” (Tillman 2021), and “Georgia-23RKN” (Branch and Brenneman 2024). Additionally, it features in the pedigree of “NemaTAM II” (Baring et al. 2023), “TifNV-HG” (Holbrook et al. 2023), and “TifJumbo” (Holbrook et al. 2024). Its RKN-susceptible sister line, TifGP-2, derived from the same parent population, serves as a research tool to explore RKN resistance mechanisms (Holbrook et al. 2012). Furthermore, “Tifguard” has also contributed to the development of 2 additional advanced germplasm lines, TifGP-3 and TifGP-4, which combine RKN resistance with late leaf spot disease resistance (Lamon et al. 2021; Holbrook et al. 2022).

The “TifNV-High O/L” variety was developed by crossing “Tifguard” with the high-oleic cultivar “Florida-07” (Gorbet and Tillman 2009). In the breeding program, F_2_ populations were screened using marker-assisted selection to identify homozygous plants for both RKN resistance and high oleic acid content (Chu et al. 2011). Selected plants were individually harvested, and their F_2:3_ progenies were evaluated for TSWV resistance and agronomic traits. Subsequent seed increase and replicated yield testing of F_3:5_ material led to the selection of the final “TifNV-High O/L” line (C1805-3-43), resulting in a high degree of genetic similarity between “Tifguard” and “TifNV-High O/L” (Holbrook et al. 2017). Due to its close genetic relationship with “Tifguard,” “TifNV-High O/L” serves as a highly informative control in comparative studies.

Despite the resistance profile of “Tifguard,” Chu et al. (2011) found approximately 6% of F_1_ hybrids involving “Tifguard” as the female parent lacked the wild chromosome segment, attributing this to mixture or outcrossing events. Although peanut is primarily a self-pollinating crop, natural hybridization can reach up to 10%, further complicating the evaluation of possible cases of resistance breakdown (Culp et al. 1968; Coffelt 1989). However, an alternative explanation merits consideration. Cultivated peanut is a segmental allotetraploid, and occasional multivalent formation and homoeologous exchange during meiosis can generate unbalanced genomic configurations such as AAAA, AAAB, ABBB, BBBB, and deletions. In our recent studies of peanut genome stability, such large-scale chromosomal alterations have been observed in up to ∼5% of pure pedigree peanut plants and at higher frequencies in resynthesized allotetraploids (Lamon et al. 2025a, 2025b, 2026). In principle, these events could affect any major-effect locus, including the *A. cardenasii*-derived segment on chromosome A09 that confers RKN resistance.

In this study, we investigated whether homoeologous exchange or other forms of genetic instability affect the *A. cardenasii*-derived chromosomal segment on A09, which confers RKN resistance in “Tifguard.” We analyzed the genomic composition of lineages derived by single-seed descent from 10 asymptomatic and 32 symptomatic “Tifguard” plants, all collected from foundation seed grown in an RKN-infested field. These were compared with “TifNV-High O/L” and other control varieties.

## Materials and methods

### Plant material

**Fig. 1.**
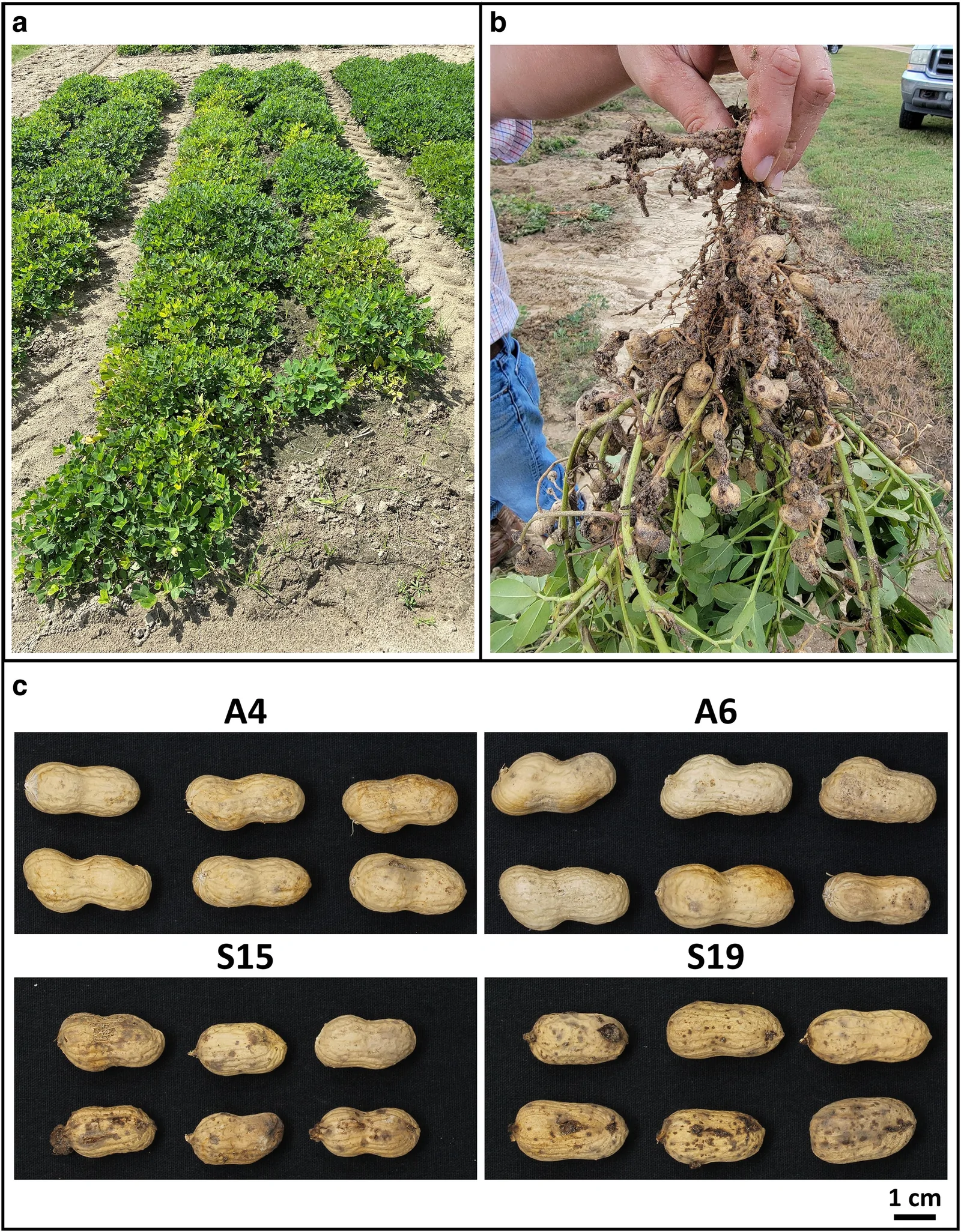
Phenotypic variation among “Tifguard” foundation seed genotypes under root-knot nematode pressure. a) Shows “Tifguard” plants displaying chlorosis and stunting, which are characteristic symptoms of root-knot nematode infestation. b) Highlights the root system of a symptomatic “Tifguard” foundation seed genotype grown in 2021 in the nematode-infested field at Black Shank Farm, Tifton, Georgia (source: Prof. Tim Brenneman). c) Illustrates pods from selected asymptomatic (A4 and A6) and symptomatic (S15 and S19) “Tifguard” plants collected from the same nematode-infested field.

In 2021, Alabama “Tifguard” foundation seeds (lot #G95-411) were planted in an RKN-infested field at Black Shank Farm in Tifton, GA, which has been under continuous peanut cultivation for many years. Before harvest, plants were visually inspected for chlorosis (yellowing) and stunting (Fig. 1a), allowing the identification of both symptomatic and asymptomatic plants (Fig. 1b). From this inspection, 10 asymptomatic (A1 to A10) and 32 symptomatic plants (S1 to S32) were collected from the same field area (Fig. 1c). Lineages from each of the 42 selected “Tifguard” plants were advanced in a pollinator-free greenhouse using single-seed descent. Lineages were tracked with a consistent naming convention: for example, in “A1.1,” the “A1” indicates RKN-asymptomatic field plant 1, the single dot represents the first generation of selfing, and the following number denotes the seed number. This format was maintained across all generations. In addition to the “Tifguard” lineages, pods from 30 peanut varieties representing the 4 major market types (Runner, Spanish, Valencia, and Virginia) were collected from various sources and used as control materials (Supplementary Table 1). For each control variety, 3 individual plants were grown and analyzed in parallel with the “Tifguard” lineages.

### Genotyping and sequencing

Seeds of the 42 “Tifguard” lineages and controls were grown in the greenhouse. DNA was extracted from young leaves using the DNeasy Plant Mini Kit (Qiagen, Hilden, Germany). Genotyping was performed with the Axiom *Arachis* 48K SNP array v2 (Affymetrix, Santa Clara, California, USA) (Pandey et al. 2017; Korani et al. 2019). Additionally, 4 second-generation “Tifguard” lineages were selected for whole-genome sequencing: 2 derived from asymptomatic plants (A4.1.1 and A6.1.1) and 2 derived from symptomatic plants (S15.1.1 and S19.1.1). DNA was extracted using the same protocol and sequenced at approximately 3× coverage using Illumina NovaSeq PE150 (Novogene Co., Sacramento, California, USA).

### Genotypic data analysis

Genotypic data from the Affymetrix Axiom *Arachis* 48K SNP array v2 were analyzed in RStudio (Posit Team 2025). SNP markers were scored on a tetraploid scale (0 to 4), representing allele dosages in segmental allotetraploid peanut, using the “fitPoly” (Voorrips et al. 2011; Voorrips and Gort 2018) package (Supplementary Table 2). One symptomatic “Tifguard” lineage, S30.1, was excluded because it failed quality control. Markers with dosage calls across all individuals were retained and aligned to the *A. ipaënsis* genome (gnm1), yielding 8,968 SNPs used for all population-structure analyses. Because A- and B-subgenome chromosomes in peanut are highly syntenic, chromosomal positions are reported using the conventional A-subgenome nomenclature (e.g. A09) to refer to the corresponding homoeologous chromosome pair.

To evaluate potential seed mixtures among symptomatic “Tifguard” lineages, 2 separate discriminant analyses of principal components (DAPC) were conducted using the “adegenet” package (Jombart and Ahmed 2011). The first analysis assessed group assignment at the market type level, with samples classified into Runner, Spanish, Virginia, and Valencia types. Asymptomatic “Tifguard” lineages were included as controls within the Runner group. The second analysis focused on finer resolution within the Runner type, testing assignment to 9 specific varieties: “Georgia-06G” (Branch 2007), “FloRun ‘331’,” “Georgia-16HO” (Branch 2017), “Georgia-18RU” (Branch 2019), “Georgia-20VHO” (Branch 2021), “Georgia-14N” (Branch and Brenneman 2015), “TifNV-High O/L,” “Tifguard” (asymptomatic only), and “TUFRunner ‘297’” (Tillman 2018).

In both DAPC analyses, the number of retained principal components (PCs) was optimized using cross-validation with 1,000 bootstrap replications via the xvalDapc() function, applied only to control samples to reduce overfitting and ensure stable assignment probabilities. Once the optimal model was selected, symptomatic “Tifguard” lineages were treated as supplementary individuals. In this framework, supplementary individuals do not contribute to the construction of the discriminant functions but are projected onto the axes derived from the training dataset. This approach allows group prediction for individuals with unknown or uncertain classification, provided they are genotyped at the same loci. Their coordinates are computed using the same centering, scaling, and discriminant coefficients as the contributing individuals.

To determine the contribution of chromosome A09 to the observed differentiation between symptomatic and asymptomatic lineages, a neighbor-joining tree was constructed using Prevosti's absolute genetic distance (Prevosti 1974; Prevosti et al. 1975) with 1,000 bootstrap replicates excluding markers assigned to chromosome A09. The analysis was conducted using the “adegenet,” “ape” (Paradis and Schliep 2019), and “poppr” (Kamvar et al. 2015) packages. To further investigate the role of chromosome A09, SNPs specific to the *A. cardenasii*-derived resistance segment were used to compare allele dosages across symptomatic and asymptomatic “Tifguard” lineages and the “TifNV-High O/L” control (Bertioli et al. 2021a).

### Sequencing data analysis

Sequencing data analysis of reads from symptomatic and asymptomatic “Tifguard” lineages followed a procedure similar to that described by Lamon et al. (2025a). Reads were processed to filter out low-quality reads, trim low-quality bases, and remove adapter sequences using BBMap version 39.01 (Bushnell 2014). An *in silico* synthetic tetraploid peanut genome was created by combining reads of the *A. duranensis* (AA-genome) and *A. ipaënsis* (BB-genome) genomes version gnm1 (Bertioli et al. 2016). The filtered reads from the 4 “Tifguard” lineages were aligned to the synthetic tetraploid peanut genome using BWA (version 0.7.17-r1188) (Li and Durbin 2009). Mapped read depths at each genomic position were normalized by dividing the observed depth by the expected coverage. The normalized values were summarized in 10 kb bins and plotted using “ggplot2” (Wickham 2016) in RStudio.

## Results

### Seed mixture identification using DAPC

**Fig. 2.**
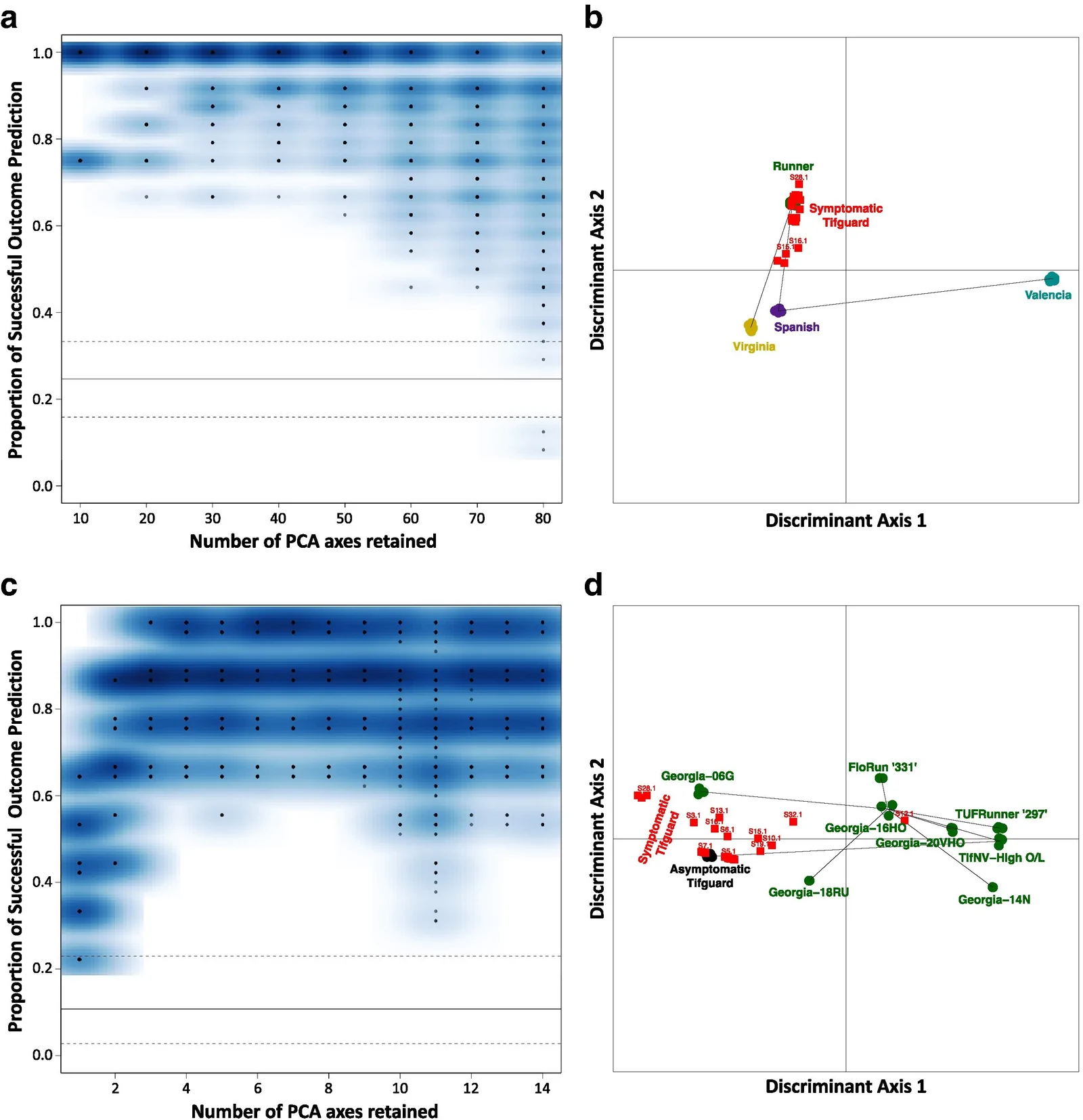
Genetic differentiation of symptomatic “Tifguard” lineages by discriminant analysis of principal components (PCs). Discriminant analysis of principal components (DAPC) was performed at 2 hierarchical levels: market type (a, b) and Runner varieties (c, d). a) and c) show cross-validation results (1,000 replicates), with the number of retained PCs on the *x* axis and the proportion of successful outcome prediction on the *y* axis. b) and d) show the corresponding DAPC scatterplots based on the selected number of PCs. Sample groups are indicated as follows: asymptomatic “Tifguard” lineages (black dots), symptomatic “Tifguard” lineages (red squares), other Runner varieties (green dots), Spanish (purple), Valencia (cyan), and Virginia (yellow). In b), asymptomatic “Tifguard” lineages appear in green because they were grouped with Runner controls at the market type level.

At the market type level, cross-validation of the DAPC model (1,000 replicates) indicated that retaining 40 PCs minimized the root mean squared error (RMSE = 0.050). Because RMSE increased beyond 40 PCs, indicating overfitting, 40 PCs were retained for subsequent DAPC analyses (Fig. 2a). DAPC clearly separated the 4 major peanut market types along the first 2 discriminant functions. The first axis primarily distinguished Spanish, Valencia, and Virginia types, whereas the second axis separated Runner types from the remaining groups (Fig. 2b). All symptomatic “Tifguard” lineages clustered with the Runner-type controls and were assigned accordingly, indicating no evidence of seed mixture at the market type classification level.

At the Runner variety level, cross-validation (1,000 replicates) indicated that 6 to 7 PCs fell within the range of minimum RMSE values (minimum RMSE = 0.135 at 7 PCs). Because error increased beyond this range and 6 PCs provided clearer separation among varieties, 6 PCs were retained for DAPC analyses (Fig. 2c). DAPC successfully resolved 9 Runner varieties, with asymptomatic “Tifguard” lineages forming a distinct cluster clearly separated from other Runner genotypes along both discriminant axes (Fig. 2d). Most symptomatic “Tifguard” lineages clustered near the asymptomatic “Tifguard” controls, consistent with a shared genetic background. However, 8 lineages were assigned to different varieties. Specifically, S3.1, S4.1, S13.1, S28.1, and S31.1 were assigned to “Georgia-06G”; S10.1 and S32.1 to “Georgia-18RU”; and S12.1 to “Georgia-16HO.” All DAPC assignments were strongly supported. These results indicate that susceptibility is not due to seed mixture across market types but reflects either mixture within the Runner group or variation within the “Tifguard” genetic background.

### Contribution of chromosome A09 to differentiation between symptomatic and asymptomatic “Tifguard” lineages

**Fig. 3.**
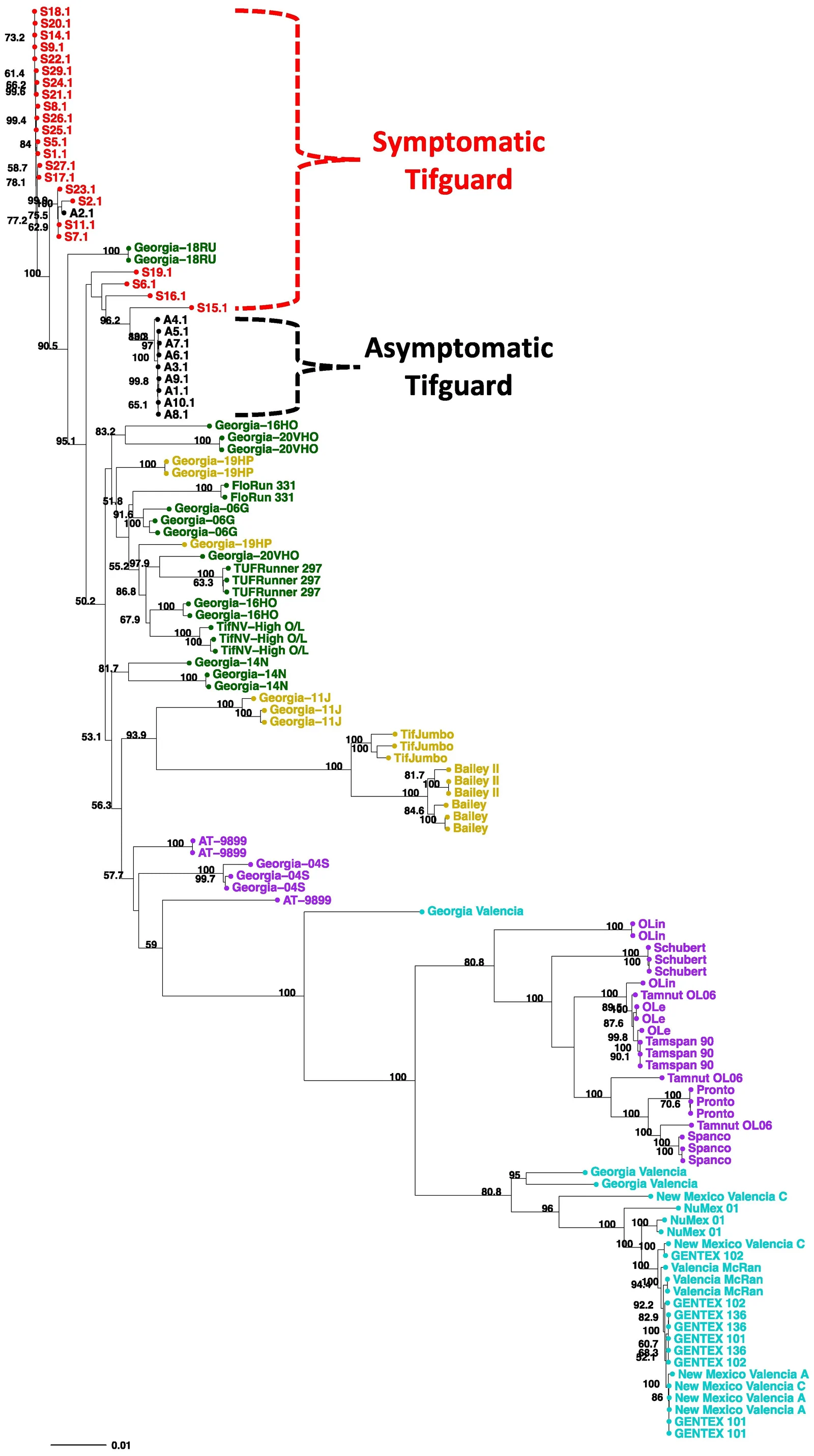
Phylogenetic clustering of symptomatic and asymptomatic “Tifguard” lineages after excluding chromosome A09. Phylogenetic tree was constructed using SNP markers excluding chromosome A09. Asymptomatic “Tifguard” lineages (black), symptomatic “Tifguard” lineages (red), and other market types, Runner (green), Virginia (yellow), Spanish (purple), and Valencia (cyan), are shown. Asymptomatic “Tifguard” lineages cluster close to symptomatic lineages, particularly S6.1, S15.1, S16.1, and S19.1, highlighting the role of chromosome A09 in their genetic separation.

To assess the contribution of chromosome A09 to genetic differentiation between symptomatic and asymptomatic “Tifguard” lineages, an unrooted neighbor-joining phylogenetic tree was generated using 1,000 bootstrap replicates, excluding markers assigned to A09. In this tree, asymptomatic “Tifguard” lineages formed a compact, well-supported cluster with low genetic divergence and were positioned close to the symptomatic lineages (Fig. 3). The only exception was A2.1, which grouped with the symptomatic cluster. The asymptomatic lineages were clearly separated from “TifNV-High O/L” and “Georgia-14N” but showed moderate genetic proximity to “Georgia-19HP.” “TifNV-High O/L,” “Georgia-19HP,” and “Georgia-14N” are RKN-resistant cultivars. Both “TifNV-High O/L” and “Georgia-19HP” were derived from “Tifguard,” whereas “Georgia-14N” was not derived from “Tifguard” but carries the same introgressed resistance segment originally introduced from “COAN.” Overall, these results reinforce the central role of chromosome A09 in differentiating symptomatic and asymptomatic “Tifguard” lineages.

### Genotyping and sequencing do not support homoeologous exchange on chromosome A09

Dosage scores for SNP markers within the *A. cardenasii*-derived segment on chromosome A09, which is known to confer resistance to RKN, were analyzed across 3 groups: “TifNV-High O/L” control samples, symptomatic “Tifguard” lineages, and asymptomatic “Tifguard” lineages (Supplementary Table 3). All asymptomatic “Tifguard” lineages, with the exception of A2.1, showed dosage scores identical to those observed in the “TifNV-High O/L” control samples, supporting the presence of the resistance-associated chromosomal segment. In contrast, all symptomatic lineages displayed a different but consistent pattern, indicating the absence of the introgression. The A2.1 dosage scores matched those of the symptomatic group. This suggests that A2.1 likely lacks the resistance-conferring segment but was mistakenly included among the asymptomatic lineages due to an absence of visible RKN symptoms in the field, possibly resulting from low nematode pressure or escape from infestation.

To investigate whether the dosage differences could be attributed to homoeologous exchange or other forms of genetic instability, 2 representative asymptomatic lineages (A4.1.1 and A6.1.1) and 2 symptomatic lineages (S15.1.1 and S19.1.1) were selected for whole-genome sequencing. Both groups showed unbalanced genomic regions consistent with homoeologous exchange in several locations, including the bottom of chromosome A02/B02, A04/B04, and A06/B06, and the top of chromosome A05/B05 (Supplementary Figs. 1–4). These regions are commonly found in cultivated peanut and are not specific to RKN resistance (Bertioli et al. 2019).

**Fig. 4.**
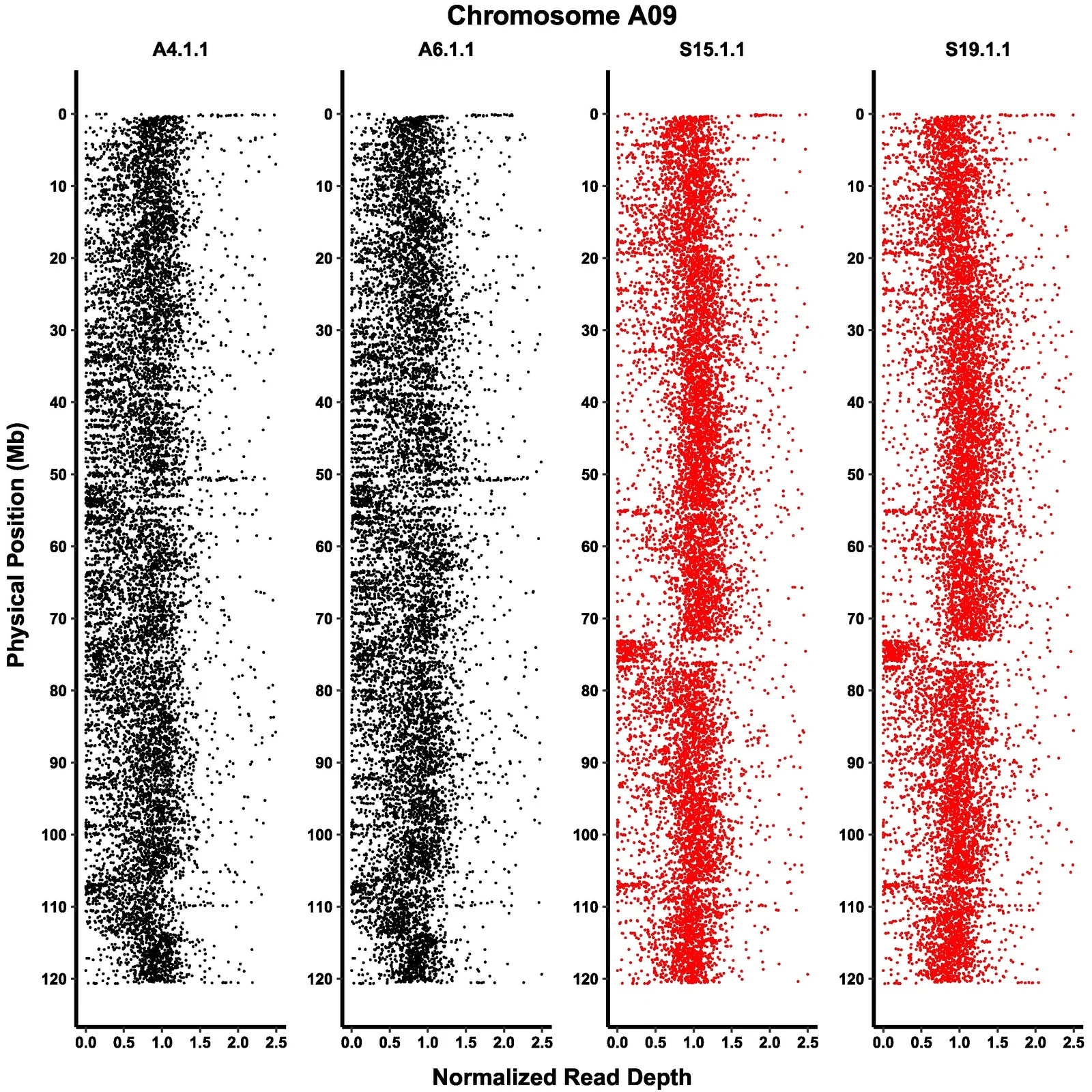
Chromosome A09 read-depth profiles in asymptomatic and symptomatic “Tifguard” lineages. Chromosome A09 read depth profiles based on 10 kb bins from asymptomatic “Tifguard” lineages (A4.1.1 and A6.1.1, black) and symptomatic lineages (S15.1.1 and S19.1.1, red) aligned to *Arachis duranensis* chromosome 09. All genotypes show an average normalized read depth near 1, indicating overall genomic stability. Asymptomatic lineages display broader read depth variability, consistent with the presence of an *A. cardenasii* introgression that reduces alignment efficiency to the *A. duranensis* reference. Symptomatic lineages show mapping patterns similar to other *A. hypogaea* of pure pedigree.

Chromosome A09, however, revealed distinct differences (Fig. 4). In both symptomatic and asymptomatic lineages, the majority of normalized read depth values were centered around 1, indicating no widespread genomic imbalance or signs of homoeologous exchange on this chromosome. Nevertheless, the asymptomatic lineages exhibited a broader range of values with many scores between 0 and 1. This pattern indicates the presence of a large *A. cardenasii* introgression, which reduces mapping efficiency to the *A. duranensis* reference genome. In contrast, symptomatic lineages showed a uniform distribution centered near 1 across chromosome A09, consistent with retention of the complete endogenous *A. hypogaea* A09 region at the expected dosage and absence of the *A. cardenasii* introgression.

Taken together, these findings show that asymptomatic “Tifguard” lineages carry a large *A. cardenasii* introgression on chromosome A09, whereas symptomatic lineages do not. The differences in normalized read depth support the association between this structural variant and RKN resistance. Since both groups exhibit similar patterns of homoeologous exchange on other chromosomes, the variation in resistance corresponds to the presence or absence of the A09 introgression.

## Discussion

“Tifguard” is a Runner-type peanut cultivar developed through single-seed descent and is widely recognized for its dual resistance to TSWV and RKN, the latter conferred by a large *A. cardenasii*-derived segment of chromosome A09. This segment, introduced into cultivated peanut's genetic pool via the induced allotetraploid TxAG-6 (Simpson et al. 1993) and subsequently through the cultivar “COAN” (Simpson and Starr 2001), has been extensively used in breeding programs to develop RKN-resistant varieties. Despite its resistance profile, approximately 6% of F_1_ hybrids involving “Tifguard” as the female parent were reported as susceptible to RKN 3 years after its release (Chu et al. 2011).

There are several plausible explanations for the presence of RKN-symptomatic individuals in “Tifguard” populations: seed mixture, natural hybridization, and genetic instability. The first 2 are straightforward. Seed mixture can occur during cultivar development and seed multiplication, while cross-pollination by bees has been reported at rates of up to 10%, even though peanut is predominantly self-pollinating (Culp et al. 1968; Coffelt 1989). The third explanation would previously have been considered unlikely; however, recent work has shown that cultivated peanut exhibits spontaneous large-scale chromosomal abnormalities at frequencies of approximately 1% to 5%, including allele dosage changes and deletions (Lamon et al. 2025a, 2025b, 2026).

To investigate whether genomic instability could explain the observed field symptoms, we analyzed 42 “Tifguard” lineages derived from plants grown in an RKN-infested field, focusing on the integrity of the wild-introgressed segment on chromosome A09. Our analyses revealed no signs of homoeologous exchange, copy-number imbalance, or deletions affecting chromosome A09 in the symptomatic lineages. Both SNP array and whole-genome sequencing data showed that symptomatic lineages lack the *A. cardenasii* introgression and instead retain the endogenous A09 region at the expected A-subgenome copy number. Thus, differences in resistance are associated with the simple presence or absence of this segment, rather than with structural instability.

Among the symptomatic lineages genotyped, 8 were consistent with mixture from other cultivars, based on DAPC analyses. Although the lineages in this study were derived from foundation seed, the sampling strategy was intentionally biased toward visibly symptomatic plants, likely increasing the chance of detecting seed mixtures.

The remaining 23 symptomatic lineages lacked the A09 resistance segment. In the neighbor-joining tree constructed without A09 markers, these 23 lineages clustered closely with the asymptomatic “Tifguard” group, indicating a largely shared genetic background across the rest of the genome (Fig. 3). Notably, 4 of these symptomatic lineages were positioned immediately adjacent to the asymptomatic cluster, differing primarily at the resistance locus. This pattern indicates that the key genomic distinction between these groups is the presence or absence of the *A. cardenasii* introgression, rather than broader genome-wide divergence.

Altogether, our results indicate that the RKN infestation symptoms observed in “Tifguard”-derived lineages are explained by seed mixture and possibly cross-pollination. Unlike polygenic resistance, major-effect traits behave as binary features: presence of the segment confers resistance, while its absence results in full susceptibility. Consequently, even rare seed mixture or cross-pollination events have disproportionate phenotypic impact. This places unusually high demands on genetic and seed purity when deploying and maintaining cultivars carrying major-effect traits.

Standard checks of cultivar identity can confirm the expected cultivar background, but they do not directly test if the A09 segment required for RKN resistance has been retained. Although genetically modified crops differ from conventionally bred peanuts in how the target traits are introduced and regulated, the practical issue is similar: cultivar value depends on maintaining the genetic feature responsible for the target trait across generations. Routine marker-based testing at breeder, foundation, registered, and certified stages would directly verify trait retention. The declining cost of DNA marker analysis makes such testing increasingly practical.

## Data Availability

Whole-genome sequencing data can be accessed through NCBI under BioProject accession PRJNA1240330. Sequencing analysis scripts are available at https://github.com/brianabernathy/ABGD. Supplementary Figures and Supplementary Tables are available at FigShare, G3 (Bethesda) online (DOI: 10.25387/g3.32826953).
